# Medicinal plants of Dagala region in Bhutan: their diversity, distribution, uses and economic potential

**DOI:** 10.1186/s13002-016-0098-7

**Published:** 2016-06-24

**Authors:** Phurpa Wangchuk, Kuenga Namgay, Karma Gayleg, Yeshi Dorji

**Affiliations:** Queensland Tropical Health Alliance, Australian Institute of Tropical Health and Medicine, James Cook University, Cairns campus, QLD 4878 Australia; Policy and Planning Division, Ministry of Agriculture and Forest, Thimphu, Bhutan; National Traditional Medicine Hospital, Department of Traditional Medicine, Ministry of Health, Thimphu, Bhutan; Personal Physician of Je Khen Po (Religious Head of Bhutan) and the Ex-consultant of Menjong Sorig Pharmaceuticals, Ministry of Health, Thimphu, Bhutan

**Keywords:** Bhutanese *g.so-ba-rig-pa* medicine, Menjong Sorig Pharmaceuticals, Dagala Gewog, Medicinal plants

## Abstract

**Background:**

The traditional *g.so-ba-rig-pa* hospitals in Bhutan uses more than 100 polyingredient medicines that are manufactured by the Menjong Sorig Pharmaceuticals (MSP). The MSP has been collecting medicinal plants from Lingzhi region for about 48 years and therefore the ecological pressure on these plants have increased. It is MSP’s top priority to identify an alternative collection site to ease the problem. Therefore, this study was carried out to determine whether Dagala region could potentially be an alternative collection site for MSP.

**Methods:**

First the multidisciplinary research team generated a tentative plant list by reviewing a body of ancient *g.so-ba-rig-pa* literature, current formulations, and the MSP medicinal plants inventory documents. Second, the research team visited the study areas in Dagala region for spot identification of medicinal plants. Third, we confirmed our traditional and botanical identification by crosschecking the descriptions with the series of books on traditional texts, Flora of Bhutan, scientific papers on medicinal plants, and the plant databases.

**Results:**

We have identified 100 species of high altitude medicinal plants from Dagala region. Of these, 24 species grow abundantly, 29 species grow in moderate numbers and 47 species were scarce. More than 85 species belonged to the herbaceous life form and 51 of them are used as a whole plant. A total of 68 species grow in between 4000 and 4999 meter above sea level. These 100 medicinal plants represented 39 different families and 80 genera and the maximum number of plants belonged to the family Asteraceae. Of 60 species that are currently used for formulating medicines at MSP, 16 species have economic importance with potential for commercial collection. Out of seven areas covered by the survey, Kipchen hosted maximum number of medicinal plants (21 species).

**Conclusions:**

Our survey identified 100 medicinal plants from Dagala region and of these, 16 species has economic potential that could benefit both MSP and Dagala communities. It is feasible to establish an alternative medicinal plants collection center in Dagala Gewog.

## Background

**Fig. 1:**
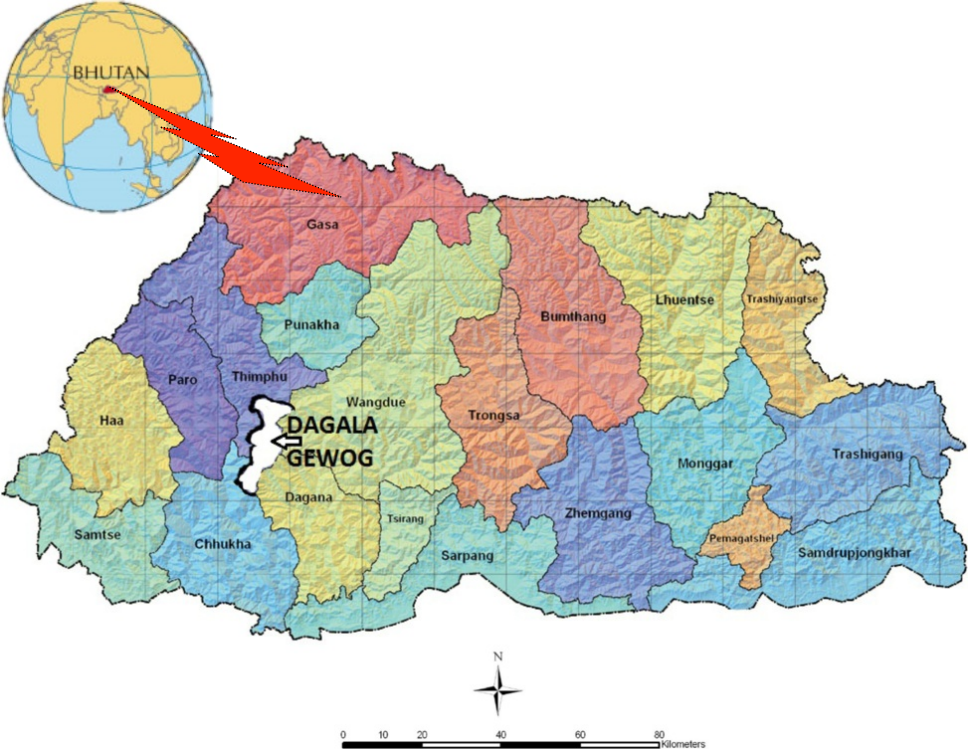
Map of Bhutan and Dagala Gewog (shaded white and labeled with white arrow) [21]

Bhutan has a total of 20 Dzongkhags (districts) and 205 Gewogs (blocks). Dagala, also known as Dakarla, is one of the eight Gewogs under Thimphu Dzongkhag with total land coverage of 85 km^2^, five chiwogs (villages), 178 households and 814 inhabitants [1] (Fig. 1). It has huge rangelands and supports livestock including the Yaks. People inhabiting these areas are known as *Byjops or Jops* and live in the scattered settlements at widely differing altitudes as yak herders or looking after the cattle. Yaks give milk, butter, cheese, buttermilk, *chugo* (dried hardened cheese consumed as snacks), tough hairy wool, meat and dung. Its tail is traded as a spiritual item, decoration peace and house duster. Its hairy wool is used for weaving clothes, bags, tents, ropes and handicraft items. Its dung serves as manure and is often used as a substitute for firewood. They barter or sell their wool, yak’s meat (expensive than normal beef) and other dairy products for food grains, sugar, tea leaf, clothing and other modern merchandise from the city.

Topographically, Dagala falls within Wang River Basin (WRB) and is known for its beautiful snow-capped mountains, ridges, alpine pastureland (*tsamdro*), beautiful lakes, rivers and pristine environment that are still intact in its natural forms. It is said that there exist countless mountains that connects Lhasa (Tibet/China) in the north and India in the south [2, 3] and these mountains are believed to contain as many as 108 lakes [2]. The lakes supports fish and other lake ecosystems and also serve as the perennial river sources, which attracts rich fauna and flora downstream. There were few unsubstantiated claim about the lush growth of medicinal plants in Dagala region. It is possible that this claim are true as it share similar topography, vegetation and climatic conditions as that of Lingzhi region in the northern part of the country where medicinal plants thrive plentifully. Lingzhi region has been the medicinal plants collection centre for the Ministry of Health (MoH) in Bhutan for more than 48 years. The MoH, under the instruction of the third King of Bhutan, Jigme Dorji Wangchuck, integrated the traditional *g.so-ba-rig-pa* (pronounced as *So-wa-rig-pa*) medicine with the mainstream modern health care system in 1967 [4, 5]. The Bhutanese *g.so-ba-rig-pa* medicine (BSM) belong to the larger corpus of a scholarly Tibetan medicine, which is in practice wide across the world [6]. In ancient times, *g.so-ba-rig-pa* medicine was only practiced in Tibet, Bhutan, Nepal (Dolpo and Mustang), Mongolia, and India (Sikkim and Ladakh).

The *g.so-ba-rig-pa* medicine has undergone constant process of evolution, adaptation, scientific scrutiny and quality control system ever since its integration with modern health care system in 1967 [4, 5, 7–18]. The BSM has evolved into a more sophisticated system with three functional organizations: a) National Institute of Traditional Medicine - recently transformed into a Faculty of Traditional Medicine (FTM) under the Khesar Gyalpo University of Medical Sciences, b) National Traditional Medicine Hospital - recently converted to a Department of Traditional Medicine DTM) under MoH, and c) Pharmaceutical and Research Unit - currently renamed as Menjong Sorig Pharmaceuticals under MoH. While the FTM provides university level training and education to the students, the DTM oversees the provision of free traditional health care services to the people through 58 traditional medical hospitals/units in the country, which are established alongside modern hospitals and Basic Health Units. After completing the Drungtsho course (5 years duration) and Menpa course (3 years duration) from the FTM, the students are recruited by the Royal Civil Service Commission of Bhutan to serve in the traditional medicine hospitals/units and the Menjong Sorig Pharmaceuticals (MSP).

The MSP manufactures more than 100 different polyingredient medicinal formulations and supplies them to the traditional medicine hospitals and units across the country [19]. The polyingredient medicinal formulations are prepared into different dosage forms as powder, capsules, pills, tablets, ointments and decoctions. The raw materials (mostly medicinal plants) for preparing these formulations are either collected within Bhutan (mostly from Lingzhi region) or imported from India. The medicinal plants, which grow in the higher elevation of alpine mountains (>2000 meters above sea level) including that from Lingzhi region, are known as the High Altitude Medicinal Plants (HAMP) and the others that grow in the temperate and tropical environment are called the Low Altitude Medicinal Plants (LAMP) [20]. Due to persistent collections of HAMP from Lingzhi region for more than 48 years, the pressure on medicinal plants has increased significantly over the recent years. Therefore, identifying an alternative medicinal plants collection site for HAMP have been one of the MSP’s top priority. Our survey/study of medicinal plants from Dagala region is in alignment to this priority area and addresses the important research questions including: Does Dagala region host as many medicinal plants as Lingzhi region? What types of medicinal plants grow there? What is their status? Could Dagala be an alternative collection site for MSP? Could *Jops* benefits through the medicinal plants collection program? Our ethnobotanical survey findings are presented here for the first time. It serves as a case study with relevance to Bhutanese *g.so-ba-rig-pa* medicine in particular and would be of interest to the mainstream scholarly Tibetan medicine-practicing institutions (countries) including India (Sikkim, Ladakh and Dharamsala - Tibetan refugees), China (Tibet and Shangri-la County), Nepal (Dolpo and Mustang), Mongolia, Russia, USA, Austria, Switzerland (PADMA company), Spain, UK, Germany and others across the globe.

## Methods

### Study area and population

Darkarla or Dagala Gewog (often spelt as Geog, English translation is ‘block’-administratively demarcated region constituted by many villages) (Fig. 1) [21] is made of five chiwogs (villages): Chamgang Maed, Chamgang Toed, Doongdrog, Wangdrog and Gyaltala. These five villages have the total population of 814 people [1]. Dagala has the total land area of 24,608 ha (ha) with 13,646 ha under tree cover, 4125 ha under shrubs, 2049 under meadows, and 4642 under snow cover [22]. About 58.8 % of these lands lie in the Alpine geographical zone (3600–7500 meters above sea level (masl), 5.5 °C annual mean temperature, <650 mm rainfall per annum), 39.7 % under cool temperate zone (2600–3600 masl, 9.9 °C annual mean temperature, 650–850 mm rainfall per annum), and only 1.6 % under Wet-subtropical zone (600–1200 masl, 19.5 °C annual mean temperature, 1200–2500 mm rainfall per annum) [22, 23]. Dagala has grasslands/meadows, Rhododendron shrubberies and conifer forests, and is believed to contain as many as 108 lakes with only eight sighted so far [2]. These lakes are the sources of perennial rivers that support diverse fauna and flora downstream.

We have chosen Dagala as our study area for the following reasons: a) MSP is in dire need of an alternative collection centre for HAMP and Dagala presented as a viable alternative sources of medicinal plants. It is located in a close proximity to MSP and share similar agro-climatic features to that of Lingzhi region (current medicinal plants collection sites for MSP), b) there was unsubstantiated/anecdotal claim about the lush medicinal plants growth in the region, c) no medicinal plants survey has been conducted in this region, and d) Dagala *Jops* are poor and their engagement in the medicinal plants collection program could help them generate cash income. We have covered seven main areas under Dagala Gewog and they are:

- Chalichung (includes Zewrinang and Panka) (4100–4180 masl)
- Dabgaythang (includes Lharigang, Thoi Puendhun and Drosinang) (4200–4220 masl)
- Hammanyi (includes Dagaytsho, Tshatsho) (4380–4980 masl)
- Kheregewa (includes Chalelha) (4020–4170)
- Kipchen (includes Gur and Pang Yumchen) (2900–3900 masl)
- Tshotshom (includes Tserigang and Byiledze) (2870–4110 masl)
- Yumtsho Gewa (includes Wathachen and Lhabhadophu/Labhatama) (4180–4290 masl)

There is no motor road in all these study sites and takes many days to reach there on foot. From some halt points, the research team had to walk or trek minimum of 1–2 days to find some of the listed medicinal plants.

### Study design, survey methods and team

Our study was to survey and identify *g.so.ba-rig-pa* high altitude medicinal plants that grow in Dagala region. It is a literature-guided observational and plant identification study. We have used the study design described by Wangchuk et al. [20]. Both traditional and botanical identification methods were used during the plant survey. In order to find out what types and numbers of medicinal plants are used in the *g.so-ba-rig-pa* medicine, we have reviewed the bodies of literature including *Shel-gong shel-phreng* (ancient textbook on *g.so-ba-rig-pa* medical system) [24], the current *Traditional Medicine Formulary of Bhutan* [19], and the medicinal plants described by Wangchuk [25] and Ugyen [26]. We have freely listed down all the HAMP that are being described in the literature and that are currently in use at MSP. MSP consider plants that grow above 2000 masl as HAMP (sometime referred to as high elevation medicinal plants (HEMP)). The plant sample size as per se was not an issue in this study, as the inventory included all the medicinal plants known to grow in Dagala region. Local *Jops* were not interviewed as they lacked knowledge on medicinal plants used by MSP. Second, the field trip was made to the study area for field observations, photographing, herbarium specimen collections and other data collections. The medicinal plants were surveyed using the convenience sampling methods. Using *g.so-ba-rig-pa* names and *g.so-ba-rig-pa* plant characterization, we did a spot identification of the medicinal plants while in the field. We have also used few Tibetan textbooks [27, 28] to assist our *g.so-ba-rig-pa* plant identifications. The location of specimen collecting areas and the altitudes were recorded using a hand-held Garmin Etrex GPS-Altimeter unit. Other standard data such as vegetation, habitat description, other medicinal plants present, local plant name, locality name (if known) and species abundance were also recorded at each field site. Herbarium specimens were pressed, prepared and preserved at the Menjong Sorig Pharmaceuticals in Bhutan.

After authenticating and confirming the *g.so-ba-rig-pa* names, botanical identification of the medicinal plants were carried out in the field and confirmed either at the base-camp or upon returning to MSP based on the herbarium specimens, plant photographs and other recorded field information including life form, parts collected, altitude and the habitat. To help us and confirm our field-based botanical identification, we used series of original publications on Flora of Bhutan [29–37] and other Himalayan plants publications [38, 39] to authenticate our botanical identifications and naming. The plant nomenclature was also confirmed through ‘The Plant List’ [40], eFloras [41], and TROPICOS [42].

The research team was comprised of one *Drungtsho* (traditional *g.so-ba-rig-pa* physician from the National Traditional Medicine Hospital), one Senior *Drungtsho* Consultant (working as a chief consultant at MSP), one senior researcher (with the background on botany and plant chemistry from MSP), and one *Byjop* (local inhabitant of Dagala region) who served us as a local guide and informant for the study areas.

### Data management, criteria setting and analysis

Traditional method of plant identification is based on physical description and the organoleptic observations such as taste, odor and the color. Each plant species were scored for their status as ‘abundant’, ‘moderate’ and ‘rare’. The plants that were found less than 10 counts or citations in the study areas (only covered by our survey) were scored as rare or available in limited number. Those plants with 10–50 counts/citations in the area at the time of the survey were scored as moderately available and those with more than 50 counts were considered abundantly available.

Live specimens were collected and pressed on the way and the halt points. The photographs of live medicinal plants were also taken during the survey. The altitude and name of the places where the medicinal plants grew were recorded in the herbarium sheet or the notebook. The type of habitat for the particular species of a medicinal plant was also observed during the survey. In order to obtain lead information on some medicinal plants, few Yak herders from each halt points were casually asked if they have come across the medicinal plants in their areas by showing the plant photos of our interest.

All the information was recorded in the herbarium sheet or in the field workbook, which remained protected from rain and other damages. The information gathered by the research team during the survey was entered into MS excel sheet, analyzed and then the data were represented using bar graph. The analysis was grouped into five categories: family diversity, plant distribution by locality and altitude, plant status and the parts used. All the medicinal plants identified in the present study were ascribed their Bhutanese *g.so-ba-rig-pa* name (written in transliteration), botanical name, family, part used and ethnomedical uses. The information was maintained with the Research and Development Section of the MSP and a report was shared with the World Health Organization.

### Study limitations

Some faraway places were not covered in this study due to the short data collection timeframe and the budget. Places like Yakla, where *Dactylorhiza hatagirea and Nardostachys grandiflora* were reported to grow plentifully could not be covered under this study.

## Results

### Habitat and diversity of medicinal plants

**Fig. 2:**
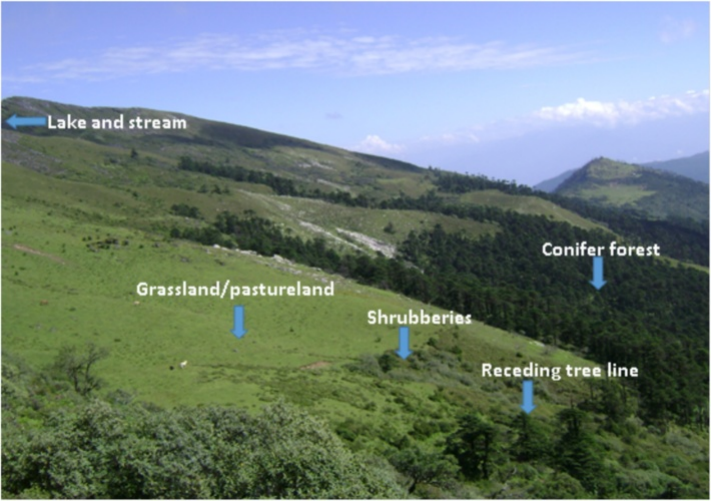
Vegetation types of Dagala region where diverse medicinal plants flourish

While Dagala *Jops* were aware of their places, we found that they were unfamiliar with the medicinal plants that grow in their forests and rangelands. The study areas had a gradient or rising vegetation with conifer forest giving way to receding tree line, rhododendron shrubberies, alpine grasslands and many lakes as we move towards higher elevations of the study areas (Fig. 2). This gradient vegetation supported various fauna and flora. Its conifer forests and Rhododendron shrubberies provides shelters to many edible mushrooms including Sangay Shamu or Matsutake (*Tricholoma matsutake*), and its alpine meadows are home to *Cordyceps sinensis* - even though we failed to source them at the time of study in August.

The areas we visited during the study period were found to host diverse range of medicinal plants belonging to at least seven habits or life forms. We have identified a total of 100 species from the seven localities of the Dagala Gewog (Table 1).

**Table 1:** List of 100 medicinal plants identified from Dagala Gewog

Latin Name [29–43]	*g.so-ba-rig-pa* name (local)	Altitude (m)	Locality	Status	Life form/Habit	Parts used	Traditional therapeutic indications [24–26, 43]
*Aconitum laciniatum* (Bruhl) Stapf (Ranunculaceae)	Bong-nga-nag-po/bdud-rtsi-lo-ma	4200	Dabgaythang	AB-U	Herb	Root	Antiseptic, analgesic, anthelmintic and reduces fluid accumulation arising from gout. Useful for leprosy and bone disorders.
*Allium macranthum* Baker (Amaryllidaceae)	ri-sgog	4180	Chalichung	A-NU	Herb	Whole	Anti-inflammatory and useful for gastritis, tuberculosis and *rlung* (air) disorders.
*Anaphalis contorta* (D.Don) Hook.f. (Asteraceae)	spra-ba	4120	Chalichung	A-U	Herb	Whole	Alleviates piles, glandular diseases and contagious infections.
*Androsace sarmentosa* Wall. (Primulaceae)	sga-tig-khra-bo	3800	Kipchen	A-NU	Herb	Whole	Febrifuge and alleviates defective serous or lymphatic fluid accumulation in the abdomen.
*Anemone griffithii* Hook.f.& Thomson (Ranunculaceae)	srub-ka	3600	Kipchen	A-U	Herb	Seed	Anticoagulant, antidropsy and helpful in regulating body temperature.
*Anemone* sp. (Ranunculaceae)	bya-rgod-srub-ka	4200	Dabgaythang	R-NU	Herb	Seed	Digestive and heals wound, snake bite and tumor.
*Arenaria kansuensis* Maxim. (Caryophyllaceae)	rtsa-a-krong	4980	Hammanyi	R-U	Herb	Flower	Useful for lung disorders and abscess formed above the chest region.
*Arisaema jacquemontii* Blume (Araceae)	dav-ba	4200	Dabgaythang	A-U	Herb	Whole	Alleviates microbial infection, swelling, malignant growth of tissues and bones, throat infection and obstruction, infertility, uterus diseases and *bad-d.kan-smug-po* disorders.
*Aster flaccidus* Bunge (Asteraceae)	lug-mig	4200	Dabgaythang	R-U	Herb	Flower	Antidote and relieves chronic bronchitis, fever, cough and cold.
*Aster neoelegans* Hand-Mazz. (Asteraceae)	yu-gu-shing-d.kar-po	3100	Tshotshom	R-U	Herb	Whole	Reduces fever arising from poisoning and heals wounds, mumps and body swelling.
*Aster stracheyi* Hook.f. (Asteraceae)	chu-de-ba	4180	Yumtsho Gewa	A-U	Herb	Whole	Treats infectious diseases, common cold and other epidemic diseases.
*Berberis aristata* DC. (Berberidaceae)	skyer-pa-dkar-po	4030	Kheregewa	AB-U	Shrub	Bark	Febrifuge, antidote and antidiarrheal. Useful for chronic cough and cold and eye disorders including conjunctivitis.
*Bistorta macrophylla* D.Don (Polygonaceae)	spang-ram	4290	Yumtsho Gewa	AB-U	Herb	Root	Antidiarrheal, antidysenteric and alleviates stomach pain.
*Boschniakia himalaica* Hook.f. & Thomson (Orobanchaceae)	stag-ma'i-yung-rdog	3092	Kipchen	A-NU	Parasitic	Seed, flower	Heals lung infections, tumor and blood disorders.
*Caltha palustris* L. (Ranunculaceae)	rta-rmig	4270	Yumtsho Gewa	R-NU	Herb	Whole	Heals wound, fractured bone including skull, ruptured blood capillary, headache, migraine and can stop profuse bleeding.
*Chrysosplenium forrestii* Diels (Saxifragaceae)	gya'-kyi-ma	4980	Hammanyi	R-U	Herb	Whole	Useful for bile diseases.
*Chrysanthemum tatsienense* Bureau & Franch. (Asteraceae)	ser-po-gzer-'jom	4170	Kheregewa	R-U	Herb	Flower	Heals fracture, common cold, chronic throat swelling and other epidemic diseases.
*Cirsium verutum* (D.Don) Spreng. (Asteraceae)	spyang-'tsher-nag-po	4040	Kheregewa	AB-NU	Herb	Whole	Emetic and purgative. Allays indigestion, swelling and *phlegm* disorders.
*Clematis acutangula* Hook.f.& Thomson (Ranunculaceae)	dbying-mong	3800	Tshotshom	A-U	Vine	Stem	Antitumor, aperitive, digestive and prevents defective body fluid accumulation.
*Codonopsis bhutanica* Ludlow (Campanulaceae)	klu-bdud-rdo-rje-nag-po	4280	Yumtsho Gewa	A-U	Herb	Whole	Allays nephrosis, numbness and tingling, gout, leprosy and helps in blood regulation.
*Corydalis crispa* Prain (Papaveraceae)	ba-sha-ka	4400	Hammanyi	R-U	Herb	Whole	Heals blood, liver and bile disorders.
*Cotoneaster microphyllus* Wall. ex Lindl. (Rosaceae)	bya-pho-tsi-tsi	3500	Kipchen	A-NU	Shrub	Seed	Alleviates bile disorders and irregular and excessive blood loss during menstruation.
*Cyananthus lobatus* Wall. ex Benth.(Campanulaceae)	sgon-bu	4180	Chalichung	R-NU	Herb	Whole	Anti-inflammatory, enemas and allays bile disorders.
*Cynoglossum wallichi* G.Don (Boraginaceae)	ne-ma-‘byar-ma	4020	Kheregewa	R-NU	Herb	Whole	Heals fractured bone, wound, and swelling.
*Dactylorhiza hatagirea* D. Don (Orchidaceae)	dbang-lag	4180	Chalichung	R-U	Herb	Root	Aphrodisiac, boost spermatogenesis and nourishes body.
*Delphinium drepanocentrum* (Bruhl) Munz. (Ranunculaceae)	bya-rkang	4300	Hammanyi	R-U	Herb	Whole	Useful for dermatitis, dysentery, wound and abscess.
*Drosera peltata* Thunb. (Droseraceae)	rtag-ngu/hoedhen	4290	Yumtsho Gewa	A-NU	Herb	Whole	Tonic, hematinic and improves sensation and sensory organs.
*Elsholtzia eriostachya* (Benth.) Benth. (Lamiaceae)	byi-ru-ser-po	4100	Tshotshom	R-U	Herb	Whole	Antibacterial. Heals wound, skin and *phlegm* disorders
*Euphorbia wallichii* Hook.f. (Euphorbiaceae)	thar-nu	3100	Kipchen	AB-U	Herb	Root	Laxative, diuretic, anti-inflammatory, purgative, useful for edema, eczema, pimple and fungal infections.
*Euphrasia officinalis* L. (Orobanchaceae)	zhim-thig-dkar-po	4200	Dabgaythang	A-NU	Herb	Whole	Antiseptic, antibacterial and useful for conjunctivitis, mouth ulcers and toothache.
*Fragaria nubicola* (Hook.f.) Lindl. ex Lacaita (Rosaceae)	bri-rta-sa-'zin	4200	Yumtsho Gewa	R-U	Herb	Whole	Anthelmintic and heals neurological and chest infections, and lung inflammation.
*Gentiana algida* Pall. (Gentianaceae)	spang-rgyan-snon-po	4290	Yumtsho Gewa	AB-U	Herb	Flower	Febrifuge and useful for sore throat.
*Gentiana crassicaulis* Duthie ex Burkill (Gentianaceae)	kyi-lce-nag-po	4180	Chalichung	R-NU	Herb	Flower	Relives fever arising from liver disorders and improves bile production.
*Gentiana tibetica* var.*robusta* (King ex Hook.f.) Kusn. (Gentianaceae*)*	kyi-lche-dkar-po	3250	Kipchen	R-U	Herb	Flower	Heals stomach and liver inflammation, wound and swelling.
*Gentiana urnula* Harry Sm. (Gentianaceae)	gang-ga-chung	4980	Hammanyi	R-U	Herb	Whole	Anti-diarrheal and detoxifier.
*Geranium procurrens* Yeo (Geraniaceae)	ga-dur	4200	Dabgaythang	R-U	Herb	Root	Antidiarrheal, antitoxin and antimalarial. Useful for cough and cold, bronchitis and the swelling of limbs.
*Geranium refractum* Edgew. & Hook.f. (Geraniaceae)	gla-sgang	4280	Yumtsho Gewa	AB-U	Herb	Root	Relieves common cough and cold. Reduces swelling of limbs.
*Gnaphalium hypoleucum* DC. (Asteraceae)	gan-da-ba-tri	4180	Chalichung	AB-NU	Herb	Root, leave, flower	Alleviates tumor, gout, kidney diseases, cough and cold, poisoning, *phlegm* and *ba-d.kan-smug-po* disorders. Used for purification rituals.
*Hemiphragma heterophyllum* Wall. (Plantaginaceae)	a-bi-ra	3900	Kipchen	AB-NU	Herb	Whole	Rejuvenate body and bodily vigor, purifies blood and maintains normal blood circulation, and alleviates gout and rheumatism.
*Heracleum candicans* var. *obtusifolium* (Wall. ex DC.) F.T. Pu & M.F. Watson (Apiaceae)	spru-na-d.kar-po	3900	Kipchen	A-U	Herb	Root	Stops bleeding (coagulant), relieves headache, and heals leprosy and neurological disorders.
*Impatiens laxiflora* Edgew. (Balsaminaceae)	byi'u-star-ga	3400	Kipchen	AB-NU	Herb	Aerial	Allays constipation, amenorrhea and difficulty in urination.
*Inula grandiflora* Willd. (Asteraceae)	ming-can-ser-po	4180	Chalichung	R-U	Herb	Flower	Heals abscess/boil, numbness, fever and evil affliction.
*Iris kemaonensis* D.Don ex Royle (Iridaceae)	dres-ma	4200	Dabgaythang	A-NU	Herb	Seed	Anthelmintic and antipyretic.
*Jaeschkea oligosperma* Knobl. (Gentianaceae)	lchags-tig	2990	Tshotshom	R-U	Herb	Whole	Heals wound, relieves common cough and cold, allays headache caused by disturbances in blood and bile.
*Juniperus pseudosabina* Fisch. & C.A. Mey. (Cupressaceae)	shug-tsher/la-shug	3780	Tshotshom	AB-U	Tree	Leaves	Alleviates kidney infections.
*Juniperus squamata* Buch.-Ham. ex D. Don (Cupressaceae)	shug-pa-tsher-can	3780	Tshotshom	AB-U	Shrub	Leaves	Alleviates kidney inflammation and reduces accumulation of defective serous fluid in the joints.
*Lepisorus contortus* (Christ) Ching (Polypodiaceae)	brag-spos-pa	3500	Kipchen	R-U	Epiphytic	Leaves	Heals bone fracture, burns, wounds and kidney disorders.
*Ligularia amplexicaulis* DC. (Asteraceae)	ri-sho	4200	Dabgaythang	R-U	Herb	Root	Emetic. Alleviates indigestion or flatulence. Heals chronic wound and contagious infections, poisoning, phlegm and *mkhris-pa* (bile) disorders,
*Lilium nanum* Klotzsch & Garcke (Liliaceae)	a-bi-kha	4200	Dabgaythang	R-U	Herb	Whole	Antidote, heals bone fracture and head injuries.
*Malcolmia africana* W.T. Aiton (Brassicaceae)	byi'u-la-phug	4200	Dabgaythang	R-NU	Herb	Whole	Antidote and helpful for indigestion, meat and food poisoning.
*Meconopsis paniculata* Prain (Papaveraceae)	ud-pal-ser-po	4200	Dabgaythang	A-NU	Herb	Whole	Useful for fever related to lung and liver disorders. Digestive and allays phlegm disorders.
*Meconopsis simplicifolia* (D.Don) Walp. (Papaveraceae)	ud-pal-ngon-po	4200	Dabgaythang	R-U	Herb	Whole	Antipyretic and antimalarial. Alleviates liver cirrhosis, lung and blood disorders.
*Nardostachys grandiflora* DC. (Caprifoliaceae)	spang-spos	4180	Chalichung	A-U	Herb	Root	Detoxifier and alleviates chronic fever and heart disorders.
*Neopicrorhiza scrophulariiflora* (Pennell) D.Y. Hong (Plantaginaceae)	hong-len	4400	Hammanyi	AB-U	Herb	Root	Useful for blood poisoning, burning sensation, heart diseases, jaundice and fever.
*Oxytropis ochrocephala* Bunge (Fabaceae)	dug-srad	3092	Kipchen	R-NU	Herb	Whole	Alleviates dropsy and neutralizes poisonous substances in the body.
*Panax pseudoginseng* Wall. (Araliaceae)	bring-gi-ra-dza	3800	Kipchen	R-NU	Herb	Root	Provides nourishment, growth, body immunity, wellbeing and longevity.
*Parmelia saxatilis* (L.) Ach. (Parmeliaceae)	sbrul-pag	3092	Kipchen	A-U	Lichen	Cap, Stalk	Heals white leprosy, sore foot and skin diseases.
*Parnassia ovata* Ledeb. (Celastraceae)	dngul-tig	4280	Yumtsho Gewa	AB-NU	Herb	Whole	Alleviates bile disorders, ganglion blockage and drug side-effects.
*Pedicularis integrifolia* Hook,f. (Orobanchaceae)	glang-snya	4180	Chalilchung	A-U	Herb	Whole	Antidiuretic, antirheumatic, regulates menstruation and heals wound.
*Pedicularis longiflora* Rudolph (Orobanchaceae)	lug-ru-ser-po	4200	Yumtsho Gewa	A-U	Herb	Whole	Alleviates coagulation, abnormal menstruation, dry mouth and tongue, and blood pressure.
*Pedicularis megalantha* D.Don (Orobanchaceae)	lug-ru-dmar-po	4020	Kheregewa	A-U	Herb	Whole	Antidote and useful for intestinal disorders.
*Pedicularis siphonantha* D. Don (Orobanchaceae)	dre-glang	3645	Kipchen	A-NU	Herb	Whole	Antidote, antidiarrheal and febrifuge for stomach disorders.
*Phlomis rotata* Benth. ex Hook.f. (Lamiaceae)	rta-lpags	4220	Dabgaythang	R-U	Herb	Whole	Strengthen broken bones, improves stiffness caused by nervous disorders, and reduces pain caused by injuries in tendons.
*Plantago depressa* Willd. (Plantaginaceae)	tha-ram	3180	Tshotshom	AB-U	Herb	Root	Antidiarrheal.
*Pleurospermum hookeri* C.B. Clarke (Apiaceae)	tang-kun-dkar-po	3980	Tshotshom	R-U	Herb	Root	Antidote, anti-inflammatory and heals heart disorders.
*Polygonatum singalilense* H.Hara (Asparagaceae)	lug-mnye	4180	Chalichung	R-NU	Herb	Flower	Improves body strength, heals wound and regulates body temperature.
*Polygonatum verticillatum* (L.) All. (Asparagaceae)	ra-mnye	4200	Dabgaythang	R-U	Herb	Root	Anthelmintic, tranquilliser, appetiser and antiaging. Reduces unwanted fluid accumulation in joints.
*Potentilla arbuscula* D. Don *(*Rosaceae)	sped-ma'-me-tog	4500	Hammanyi	AB-U	Shrub	Flower	Allays cough and cold.
*Potentilla fulgens* Wall. ex Hook. (Rosaceae)	seng-ge-bar ma	2900	Kipchen	AB-NU	Herb	Bark	Alleviates cough and cold.
*Primula* sp. (Primulaceae)	shang-dril-dkar-po	4900	Hammanyi	R-U	Herb	Flower	Febrifuge and alleviates arterial, venous, nervous, blood and air disorders.
*Primula fasciculata* Balf. f. & Kingdon-Ward (Primulaceae)	gyar-mo-thang	4280	Yumtsho Gewa	R-NU	Herb	Whole	Heals wound, inflammation and swelling.
*Primula sikkimensis* Prain (Primulaceae)	shang-dril-ser-po	3800	Kipchen	A-NU	Herb	Flower	Febrifuge, antidiarrheal (used for children) and alleviates cardiovascular disorders.
*Rabdosia rugosa* (Wall. ex Benth.) H.Hara (Lamiaceae)	zhim-thig-nag-po	4200	Dabgaythang	A-NU	Herb	Whole	Alleviates infections including eye disorders, inflammation and sudden abdominal colic pain.
*Ranunculus brotherusii* Freyn (Ranunculaceae)	lche-tsha	4200	Yumtsho Gewa	A-U	Herb	Whole	Antiseptic, antipyretic, heals wound and dries pus.
*Ranunculus tricuspis* Maxim. (Ranunculaceae)	chu-rug-pa/sbal-la	4180	Yumtsho Gewa	R-NU	Herb	Whole	Antipyretic and relieves nerve pain.
*Rheum australe* D. Don (Polygonaceae)	chu-rtsa	4100	Chalichung	AB-U	Herb	Root	Antipyretic, improves digestion, and heals wound.
*Rheum nobile* Hook. f. & Thomson (Polygonaceae)	chu-kha-metog	4980	Hammanyi	R-NU	Herb	Whole	Laxative, diuretic, antiemetic, allays swelling and fullness in the stomach and is a good re-hydration and rejuvenating agent.
*Rhododendron anthopogon* D. Don (Ericaceae)	ba-lu-dkar-po/dav-li metog	3800	Kipchen	AB-U	Shrub	Flower	Febrifuge for lung disorders and alleviates dropsy and other swelling of caused by phlegm disorders (*ba-dkan-cha-bab*). Boost immune system.
*Rhododendron glaucophyllum* Rehder (Ericaceae)	stag-ma'i-lo-ma/zhing	3820	Kipchen	AB-U	Shrub	Leaves	Hemostatic and neutralizes other toxic side effects of medicine. Also used in incense products that pacifies gods, demi-gods, deities and spirits.
*Rhododendron setosum* D. Don (Ericaceae)	ba-lu-nag-po	4110	Tshotshom	AB-U	Shrub	Flower	Allays *grang-ba* (including sexually transmitted infections) and *gag-lhog* (inflammation of the throat and muscle tissues). Used as incense for pacifying gods, demi gods, deities and spirits.
*Rosa macrophylla* Lindl. (Rosaceae)	se-rgod	3800	Kipchen	R-U	Shrub	Fruit	Antidote, heals abscess and relieves constipation, cough and cold. Also useful for liver and bile disorders.
*Salvia castanea* Diels (Lamiaceae)	jib-rtsi-chen-po/smug-po	4200	Dabgaythang	R-U	Herb	Whole	Febrifuge for toothache and liver disorders. Also heals mouth sores.
*Sambucus adnata* Wall. ex DC. (Adoxaceae)	yu-gu-shing-nag-po	3100	Tshotshom	R-U	Herb	Whole	Heals fracture, wound, poison, abscess and other skin related diseases.
*Saussurea gossypiphora* D.Don (Asteraceae)	bya-rgod-sug-pa	4480	Hammanyi	R-NU	Herb	Whole	Analgesic and alleviates blood and liver disorders.
*Saxifraga moorcroftiana* (Ser.) Wall. ex Sternb. (Saxifragaceae)	zang-tig	4220	Dabgaythang	R-NU	Herb	Whole	Antipyretic and allays bile disorders.
*Saxifraga parnassifolia* D.Don *(*Saxifragaceae)	gser-tig	4200	Dabgaythang	R-NU	Herb	Whole	Heals wouns, cough and cold, and bile disorders including jaundice.
*Scopolia lurida* (Link ex Spreng.) Dunal (Solanaceae)	thang-khrom-nag-po	4080	Kheregewa	A-U	Herb	Seed	Anthelmintic. Allays sinusitis, colic pain and microbial infections.
*Sedum ewersii* Ledeb. (Crassulaceae)	tshan-a'u-tsai	4120	Chalichung	A-NU	Herb	Whole	Alleviates fever arising from new and chronic lung infections.
*Selinum wallichianum* (DC.) Raizada & H.O. Saxena (Apiaceae)	bam-po	3100	Tshotshom	AB-NU	Herb	Seed	Allays inflammation (specific to hand, leg and stomach), and heals *skran* (tumour).
*Silene himalayensis* (Rohrb.) Majumdar (Caryophyllaceae)	ra-sug	3780	Kipchen	R-NU	Herb	Whole	Allays nasal and ear infections. Also used as cleansing (detergents) agent.
*Soroseris hookeriana*	srol-gong-ser-po	4050	Kheregewa	R-U	Herb	Whole	Relieves fever (poisoning), heals bone fracture and prevents infection and sepsis.
Stebbins (Asteraceae)							
*Spiraea arcuata* Hook.f. (Rosaceae)	Smag-shed	3000	Tshotshom	A-NU	Shrub	Leaves	Heals wound, subsides fever arising from bone infections, and drains out infected serous fluid (chuser).
*Stellaria* sp. (Caryophyllaceae)	byi-shang-dkar-mo	4100	Chalichung	AB-NU	Herb	Flower	Heals chronic fever arising due to lung disorders and tumor.
*Taraxacum officinale* F.H.Wigg. (Asteraceae)	khur-mong	4020	Kheregewa	AB-U	Herb	Whole	Removes toxin from the body (detoxifier). Allays fever arising from stomach disorders.
*Thalictrum reniforme* Wall. (Ranunculaceae)	sngo-sprin	2870	Tshotshom	A-U	Herb	Whole	Antidote, antimicrobial, antimalarial and analgesic.
*Usnea* sp. (Parmeliaceae)	dngul-skud	4190	Chalichung	A-NU	Lichen	Whole	Heals lung, liver, nerve and poison related diseases.
*Valeriana wallichii* DC. (Caprifoliaceae)	rgya-spos	4180	Chalichung	R-U	Herb	Whole	Febrifuge. Alleviates epidemic and communicable diseases, severe inflammation of the nose, throat and windpipe (trachea), swelling caused by allergens, painful spleen infections, and heals pus/abscess.
*Veratrilla baillonii* Franch. (Gentianaceae)	rgu-drus	4380	Hammanyi	R-NU	Herb	Root	Vasoconstrictor and allays wound, colic, stomachache and infectious diseases. Heals hollow organ and poisoning.
*Veronica cephaloides* Pennell (Plantaginaceae)	ldum-nag-dom-phri	4020	Kheregewa	R-U	Herb	Whole	Heals wound, ulcer and stops hemorrhage.
*Vincetoxicum hirundinaria* Medik. (Apocynaceae)	sno-dug-mo-nung	2900	Kipchen	R-NU	Herb	Whole	Febrifuge for bile disorders, anthelmintic, dysentery and throat swelling.

*NB: R-U* Rare & Used, *R-NU* Rare but Not Used, *A-U* Available & Used, *A-NU* Available but Not Used, *AB-U* Abundant & Used, *AB-NU* Abundant but Not Used

### Current status and the availability pattern of medicinal plants

**Fig. 3:**
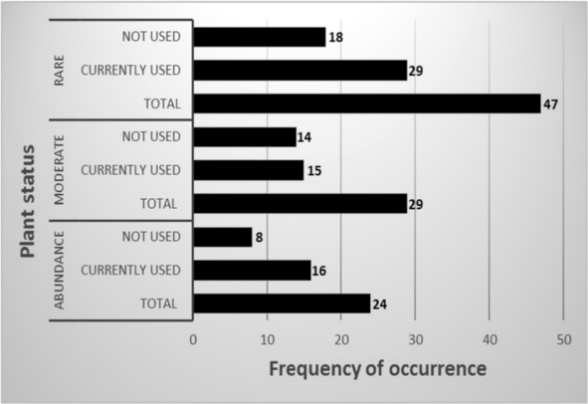
Current use and the availability status of medicinal plants

The study identified 100 medicinal plants from Dagala Gewog and 60 of them (current sourcing is from Lingzhi) are currently in use at Menjong Sorig Pharmaceuticals. Other 40 species were described in the traditional text. Upon segregation by their availability status as abundance, moderate and rare or less common (criteria described in the methods), 24 species fell within the category of abundance, 29 species under moderate and 47 species under rare or less common group (Fig. 3).

While the less commonly found species are not advisable to collect, those species that are found in abundance and in moderate distribution across the seven study sites can be collected on an annual basis. Of the 24 species that are abundantly found in Dagala Gewog, 16 of them are in current use at Menjong Sorig Pharmaceuticals (Table 1).

### Diversity of life forms or habit of medicinal plants

**Fig. 4:**
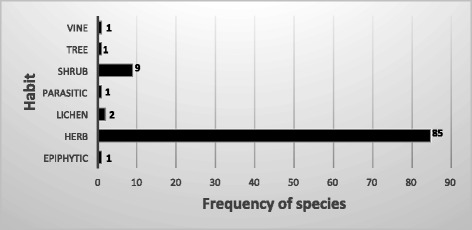
Frequency of medicinal plants per habit/life form

The 100 medicinal plants that we have identified from the study areas fell within seven habit groups as epiphytic, herb, lichen, parasitic, shrub, tree and vine (Fig. 4). Majority of the plants (85 species) belonged to the herbaceous life form. Nine plant species belonged to shrub group.

**Fig. 5:**
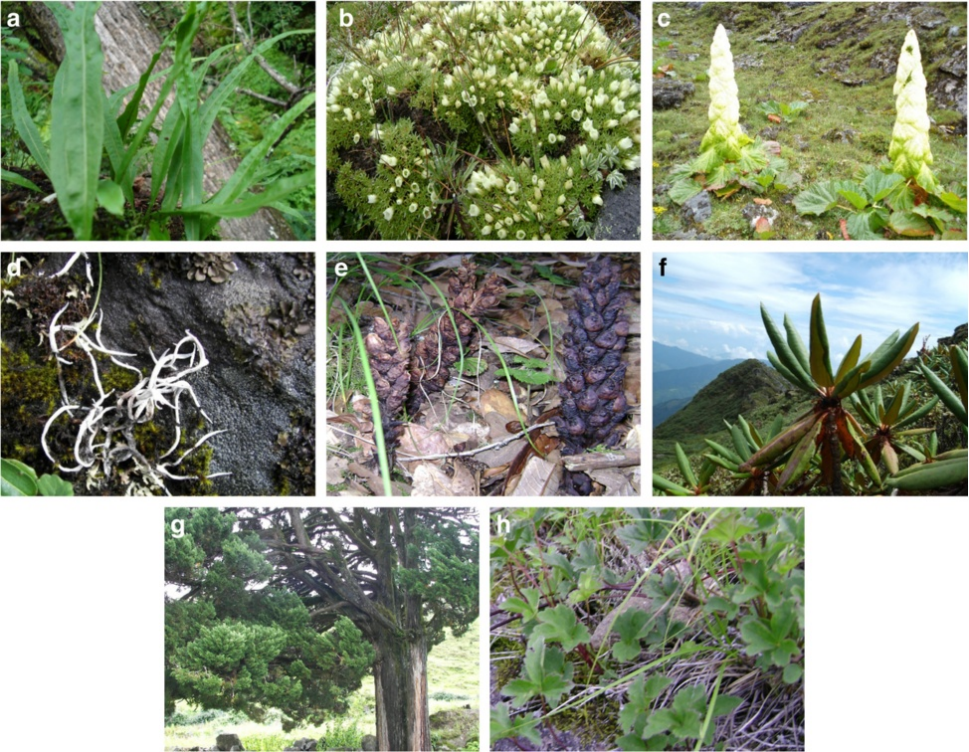
Medicinal plants representing seven different life forms (courtesy: P.W collection). *Lepisorus contortus* representing epiphytes (**a**). *Arenaria kansuensis* and *Rheum nobile* representing herbaceous form (**b**, **c**). *Usnea* species representing lichen (**d**). *Boschniakia himalaica* representing parasitic form (**e**). *Rhododendron glaucophyllum* representing a shrub (**f**). *Juniperus* species representing a tree form (**g**). *Clematis acutangula* representing a vine (**h**)

While lichens made 2 % of the medicinal plants that were identified in the study areas, the parasitic, epiphytic, tree, and vine life forms each made up 1 %. Only one tree species, *Juniperus pseudo-sabina* (*Shug-pa*) was used in the medicine and this plant is considered one of the most sought-after plants for making incense. The representative plant image of each life form is given in Fig. 5.

*Boschniakia himalaica* (Orobanchaceae) (Fig. 5e) was identified as a medicinal plant for the first time by our team (senior *Drungtsho*). The description of this plant can be found in the ancient traditional text but it is not currently used in the BTM formulation. Interestingly, *B. himalaica* is an achlorophyllic non-photosynthetic parasitic plant that grows on the roots of Rhododendrons. Since it cannot produce its own food due to lack of chlorophyll, the plant depend totally on the host (Rhododendrons) for its nourishment. The plant is similar to Squawroot (*Conopholis americana*) (also called “cancer root” or “bear cone”), which grows on a parasitic connection to the roots of oak, and also beech host trees that are found throughout eastern part of North America.

### Plant parts usage and their availability status

**Fig. 6:**
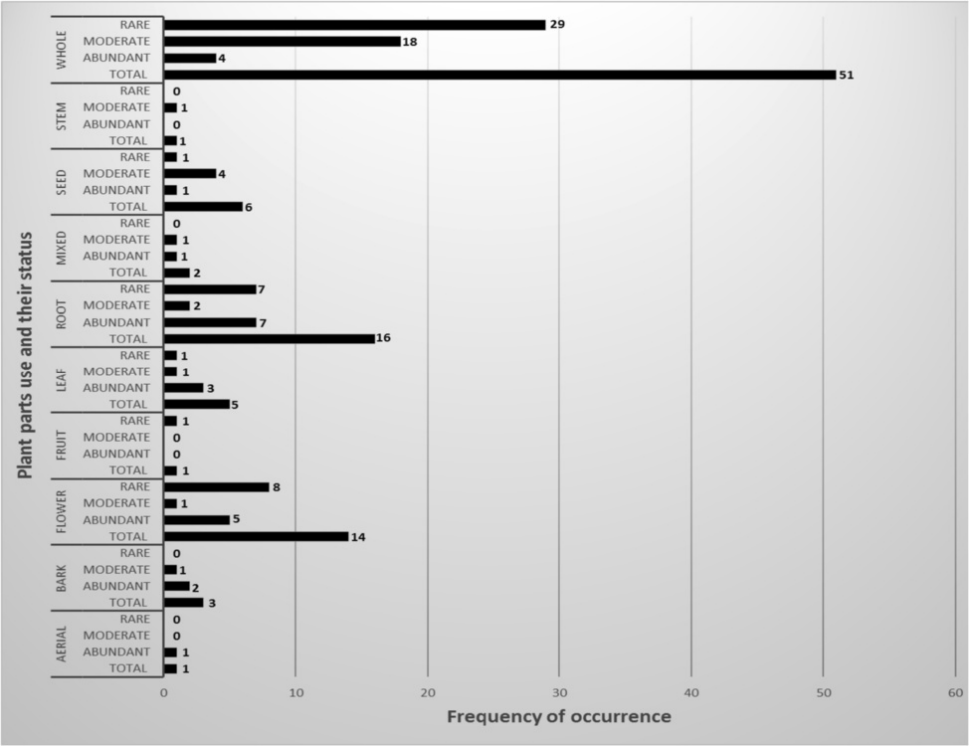
Plant parts usage category and their availability status

We found that the medicinal plants (100 species), which we identified from Dagala Gewog, are used in the Bhutanese *g.so-ba-rig-pa* medicines as a whole, stem, seed, root, leaf, fruit, flower, bark, aerial and mixed (for example, flower along with leaf) (Fig. 6).

Majority of plants are used as a whole (entire plant) with 51 out of 100 (total identified) species falling in this usage category. This agrees with the life forms present in the alpine vegetation where majority of medicinal plants belong to herbaceous life form. Out of 51 species used as a whole, 29 of them belong to the category (status) of rare or less common species (Fig 6). Sixteen species are used for its roots, which is closely followed by flower with 14 species. Aerial, stem and fruits are the least used plant parts with only one species in each usage category. A plant that is used for its fruit falls within our categorization of the plant status as rare or less commonly available medicinal plants in Dagala Gewog.

### Frequency of plant family and genera

The 100 medicinal plants that were identified from the study areas belonged to 39 families (Table 2) and 80 genera. Maximum number of plants belonged to the family Asteraceae (12 species) that is closely followed by Ranunculaceae (9 species). Gentianaceae, Rosaceae and Orobanchaceae contained 6 species each, which are closely followed by Lamiaceae, Plantaginaceae and Primulaceae with four species each. Apiaceae, Caryophyllaceae, Ericaceae, Papaveraceae Polygonaceae and Saxifragaceae all contained three species each. While six families contained two plant species each, 19 families were represented by only one species each (Table 2). Among the genera, *Gentiana* and *Pedicularis* ranked highest with each of them containing four species, which is closely followed by *Aster*, *Primula*, and *Rhododendron* with three species each. While nine genera contained two species each, all the rest of the genera contained only one plant species each.

**Table 2:** Frequency of species per plant family (Total 39 families)

Family	Frequency	Family	Frequency
Adoxaceae	1	Euphorbiaceae	1
Amaryllidaceae	1	Fabaceae	1
Apiaceae	3	Gentianaceae	6
Apocynaceae	1	Geraniaceae	2
Araceae	1	Iridaceae	1
Araliaceae	1	Lamiaceae	4
Asparagaceae	2	Liliaceae	1
Asteraceae	12	Orchidaceae	1
Balsaminaceae	1	Orobancaceae	6
Berberidaceae	1	Papaveraceae	3
Boraginaceae	1	Parmeliaceae	2
Brassicaceae	1	Plantaginaceae	4
Campanulaceae	2	Polygonaceae	3
Caprifoliaceae	2	Polypodiaceae	1
Caryophyllaceae	3	Primulaceae	4
Celastraceae	1	Ranunculaceae	9
Crassulaceae	1	Rosaceae	6
Cupressaceae	2	Saxifragaceae	3
Droseraceae	1	Solanaceae	1
Ericaceae	3		

### Distribution of medicinal plants across elevations (altitudes) of study sites

**Fig. 7:**
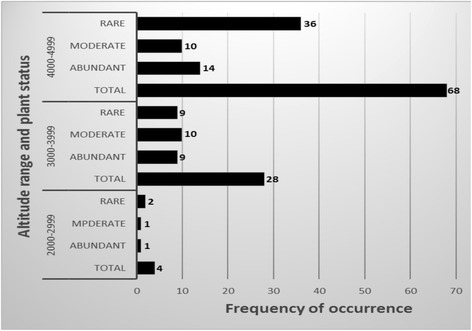
Distribution of medicinal plants across altitudes

Dagala Gewog is known for its vast alpine rangelands, lakes and steep mountain ranges with varying elevations and diverse habitat/vegetation (Fig. 2). During the field survey of medicinal plants, the lowest altitude recorded was 2870 m above sea level (masl) at Tshotsum and the highest elevation where the research team visited was 4980 masl at Hammanyi. Out of 100 medicinal plants identified from seven localities, 68 species were found growing in the altitudinal range of 4000–4999 masl (Fig. 7). In between 3000 and 3999 masl, 28 species were identified and only four species were identified from the altitudinal range of 2000–2999 masl. Analyzing the availability status of medicinal plants against each altitudinal ranges; we found that more than half of the species (36 species) identified in between 4000 and 4999 masl were rare or less commonly available in the study areas (Fig. 7). Only 14 species were found in abundance.

### Distribution of medicinal plants across seven study sites

**Fig. 8:**
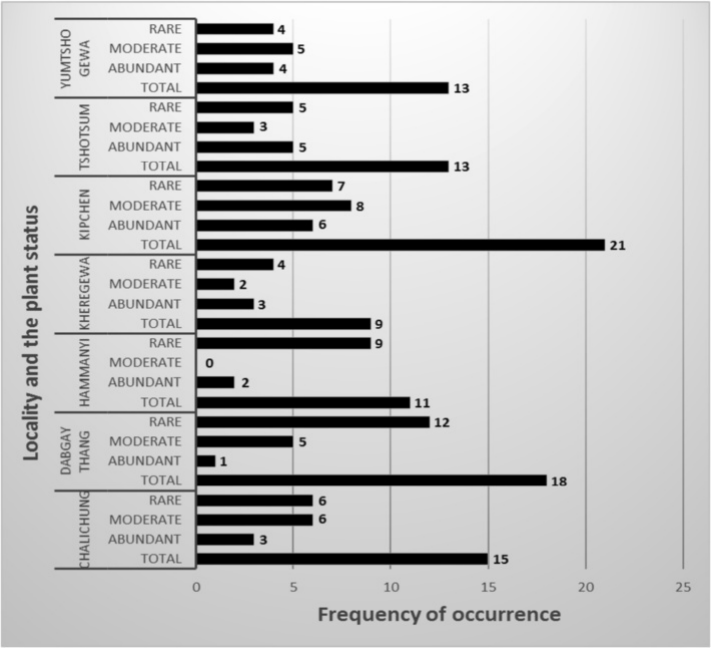
Distribution and status of medicinal plants by study sites

A total of seven major local places of Dagala Gewog were covered during the study. Among these seven study sites, Kipchen was found to host maximum number of medicinal plants with 21 species followed by Dabgaythang (18 species) and Chalichung (15 species) (Fig. 8).

While Yumtsho Gewa and Tshotsum hosted same number of species, Hammanyi had 11 species in its area. The least was found in Kheregewa with only 9 species present at the time of study. Among the localities (Fig. 8), Hammanyi and Dabgaythang hosted maximum number of less common or rare species. In Hammanyi locality, 81.8 % of the species that grow there were rare (9 out of 11 species found in the locality). In Dabgaythang locality, 66.7 % of the species that grows there were found rare (12 out of 18 species identified). Together, these two localities accounted for 21 % of the rare medicinal plants that were identified from the whole Dagala Gewog.

## Discussions

### Medicinal plants of Dagala and their economic potential

Dagala is known by its formidable mountains, vast rangelands and beautiful lakes that support rivers and fragile mountain ecosystem. Its vegetation and climatic conditions are similar to that of Lingzhi region in the north (near Tibet/China) from where MSP has been sourcing their HAMP for more than 48 years. About 116 species of HAMP have been identified from Lingzhi region [43]. This is the first report of medicinal plants identified from Dagala region in Bhutan and 60 species of the total 100 medicinal plants that we have identified from the area are currently in use (but sourced from Lingzhi in entirety) at the MSP. Out of 60 species, we have considered 29 of them as the less commonly observed species in the study areas. At least 16 species grows abundantly in Dagala Gewog and can be collected by MSP. Other species that are not currently in use at MSP adds to the inventory of already existing HAMP identified from Lingzhi region. These species can be of use in future especially when the Bhutan Traditional Medicine Essential Drug List (BTMEDL) revise and add new formulations to the list.

It is the practice of the health system and the Drug Regulatory Authority in Bhutan to revise the essential drug list after every two years to uphold Good Medicine Dispensing Practices (GMDP), keep updated on the trends and patterns of diseases, and devise appropriate pharmaceutics to combat them. When the revision of BTMEDL are made, the traditional drugs that are not used during the past two years are either deleted or substituted with alternative formulations or completely new formulations are added to the list. Most of the time these changes requires collections of medicinal plants that are not in the current list of MSP. Our study identified 40 medicinal plants that are not used for formulating any of the 100 different current formulations at MSP. However, these inventoried species will serve as the backup or contingency list for future use.

These 16 species that are found in abundance have the economic potential since MSP require them in bulk quantities to prepare *g.so-ba-rig-pa* medicines. Since *g.so-ba-rig-pa* is also practiced across the globe, these medicinal plants could be in demand by other countries including India, Nepal, Mongolia, Tibet and Switzerland (PADMA company based on Tibetan medicine). However, the first priority would be to focus on meeting the domestic demand of MSP for these medicinal plants. MSP currently engage yak herders for collecting medicinal plants from Lingzhi. As a result of medicinal plants collection program, the Lingzhip (local inhabitants of Lingzhi region) have improved their socio-economic status and contributed significantly to the realization of country’s ‘Gross National Happiness’ (GNH) indices including preservation of traditional medical knowledge, conservation of environment and socio-economic prosperity [44]. The GNH is a country’s holistic development policy propounded by the 4^th^ King of Bhutan, Jigme Singye Wangchuk and this philosophy stresses on balancing the growth of spiritual well-being and the material gains. It is an internationally well-known development philosophy. Same program could be duplicated for Dagala communities. What makes it even more promising and profitable collection site is that it is closer to MSP as compared to Lingzhi, which take 5-7 days on foot to reach there.

### Feasibility of establishing HAMP collection and drying centre in Dagala Gewog

For establishing an alternative collection site or HAMP center, we considered the following factors as critical.

- At least ten medicinal plants species must grow abundantly in the area.
- The collection site must be closest to the manufacturing section (MSP) to reduce the cost of production.
- The medicinal plants must be easily accessible for the yak herders/farmers for collection.
- The Yak herders/farmers must have interest to participate in the medicinal plants collection program.
- The collection area must have one centre-point storehouse (with drying facilities for medicinal plants) from where all the collection sites can be easily reached within one day.

Our survey found that the Dagala region meets all these five critical factors or criteria and could be an economical alternative HAMP collection centre for MSP. We have identified 16 species that grow abundantly in Dagala region. These plants have huge economic potential since MSP requires them in bulk quantities for preparing *g.so-ba-rig-pa* medicines. MSP could engage yak herders from Dagala Gewog to collect these HAMP on an annual basis. Informally socializing with the *Jops* during the survey, we came to know that they would be interested to collect the medicinal plants while tending to their regular Yak herding activities. The geographical features of the area are also favorable and easily accessible by the Yak herders. Since Kipchen host the highest number of medicinal plants and takes only 4 h walk from the motor road point in Genekha village, a medicinal plants drying center can be established there. Other places like Dabgaythang (second highest number of medicinal plants found), Hammanyi, Yumtsho Gewa and Chalichung are within 4–5 h walking distances from Kipchen. It is also only one hour drive from the MSP in Thimphu (capital city of Bhutan). Therefore, the cost of collection would be much lesser than Lingzhi. Based on these feasible factors, it is advisable for MSP to establish an alternative drying centre at Kipchen and subsequently provide training to the Yak herders (Dagala communities) on sustainable and good medicinal plants harvesting practices.

### Sustainability and management of medicinal plants

The risk factors for the sustainability of medicinal plants in Bhutan have been described elsewhere [45]. Briefly, these risk factors broadly fall into four categories: biological, ecological, social and economic factors. While Lingzhi region has been affected by all these risk factors due to persistent collection practices for more than 48 years, Dagala region provides fresh hope of sustainable supply of medicinal plants for the MSP. However, since biological (includes life form and species resilience) and ecological factors (includes habitat, population density, natural calamities, and pastureland) are inherent in nature, these risk factors are applicable to the medicinal plants that grow in Dagala region. Out of 100 species that we have identified from the study areas, 47 species can be considered rare or perceived to be lesser in number based on their population density observed at the time of the study. The risk is that some of these rare plants can be vulnerable for illegal collection and trade thereby endangering species.

**Fig. 9:**
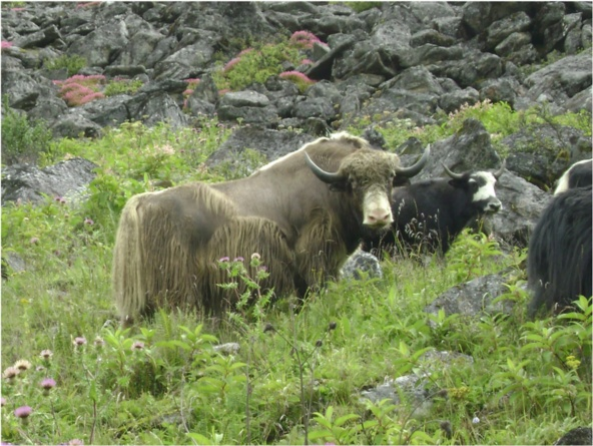
Yaks grazing the pastureland rich in medicinal plants in the Dagala Gewog

Yak/cattle herding and ecotourism are the economic mainstay of the Dagala Gewog. There is no limit to the population size of the Yaks/cattle that a family can own. Ministry of Agriculture and Forest [22] recorded that as of 2015 the percentage proportion of livestock holdings by Dagala communities included Yaks (70.9 %), poultry (12.7 %), improved cattle (10.9 %) and sheep (5.5 %). During the survey, we found that most of the Dagala family or a household, which we interacted with had minimum of 50 Yaks (Fig. 9). Overgrazing the rangelands appeared obvious to us.

Yaks consume every type of herbage vegetation and are able to feed on very short growth. They have an ability to cover large areas of grazing ground every day and could negotiate narrow footpaths on rocky slopes to consume some herbs and grasses from isolated pockets. The intensity of Yak overgrazing the alpine and temperate rangeland would accelerate the rate of ecological destruction. The pastures around Laya, Lungo, Jarila and Lingshi communities are already affected by the grazing activities of large herds of Yaks joined by flocks of wild blue sheep and marmots [46]. Most of the important HAMP are restricted to delicate, specialized and pockets of habitats. Intensive browsing or overgrazing the pastures could lead to more habitat destruction and shrinkage of the natural population sizes of the valued medicinal plants. A balance between Yaks grazing the pastureland and the growth of medicinal plants must be maintained if the medicinal plants collection program is to be established in Dagala Gewog. A study on Yaks’ food chain would shed lights on what medicinal plants are consumed by the Yaks and this could navigate the formulation of strategies to conserve those particular species.

## Conclusions and future direction

This study identified 100 species of HAMP that grows in Dagala region. Out of these at least 16 species grows abundantly and has marketing potentials for the Dagala communities. Among the places that this study covered, Kipchen was found to host maximum number of medicinal plants species. As the pressure on HAMP in Lingzhi region has been increasing due to persistent collection for more than 48 years, this finding provide basis for the MSP to establish an alternative collection center with a drying facilities at Kipchen. This place falls in the center of other localities and is not too far away from the motor road of Genekha community. Establishing an alternative HAMP collection centre in Dagala Gewog has multi-pronged benefits. The tangible and immediate benefits would include: a) Dagala communities could generate decent income through medicinal plants collection program and elevate their socio-economic status, b) MSP could obtain sustainable supply of HAMP to meet the demand of *g.so-ba-rig-pa* medicine production, c) training on sustainable collection of HAMP (always provided by MSP as a package of collection program) would educate Dagala *Jops* on the values, protection and preservation of plants, d) establishing this alternative collection center would ease the pressure on Lingzhi HAMP and could enable MSP to collect the plants on a rotational basis, and e) since Dagala region is known for eco-tourism, having the medicinal plants collection centre and the herb garden would enhance the in-flow of eco-tourists especially the botanists and the herbalists. This case study, although conducted locally in Dagala region, has international significance especially for those countries, which practices Tibetan *g.so-ba-rig-pa* medicine and uses the medicinal plants that we have identified.

## Abbreviations

BTMEDL, Bhutan Traditional Medicine Essential Drug List; GMDP, Good Medicine Dispensing Practices; GPS, global positioning system; HAMP, high altitude medicinal plants; LAMP, low altitude medicinal plants; MAP, medicinal and aromatic plants; masl, meters above sea level; MSP, Menjong Sorig Pharmaceuticals; WRB, Wang River Basin
